# Stem Cell-Based Therapies for Ischemic Stroke

**DOI:** 10.1155/2014/468748

**Published:** 2014-02-26

**Authors:** Lei Hao, Zhongmin Zou, Hong Tian, Yubo Zhang, Huchuan Zhou, Lei Liu

**Affiliations:** ^1^Department of Neurology, No.324 Hospital of PLA, Chongqing 400020, China; ^2^Department of Chemical Defense and Toxicology, Institute of Toxicology, School of Preventive Medicine, Third Military Medical University, Chongqing 400038, China

## Abstract

In recent years, stem cell-based approaches have attracted more attention from scientists and clinicians due to their possible therapeutical effect on stroke. Animal studies have demonstrated that the beneficial effects of stem cells including embryonic stem cells (ESCs), inducible pluripotent stem cells (iPSCs), neural stem cells (NSCs), and mesenchymal stem cell (MSCs) might be due to cell replacement, neuroprotection, endogenous neurogenesis, angiogenesis, and modulation on inflammation and immune response. Although several clinical studies have shown the high efficiency and safety of stem cell in stroke management, mainly MSCs, some issues regarding to cell homing, survival, tracking, safety, and optimal cell transplantation protocol, such as cell dose and time window, should be addressed. Undoubtably, stem cell-based gene therapy represents a novel potential therapeutic strategy for stroke in future.

## 1. Introduction

Stroke represents a major cause of death, followed by cancer and myocardial infarction. Its morbidity and mortality keep increasing during last decades especially in developing countries and bring severe social and economic burdens to patients and their family members. Traditional clinical management includes thrombolytic therapy, percutaneous intravascular interventions, behavioral rehabilitation strategies, and medication such as aspirin. The widely application of thrombolytic therapy is limited by the narrow time window (within 3–4.5 h after acute stroke onset) and serious hemorrhagic complication [1–3]. Percutaneous intravascular interventions usually need expertise in emergency manipulation, and a series of relative risks exist. Despite active therapies as available above, many patients suffering from stroke often remain disabled and have to rely on natural or forced rehabilitation. The high morbidity and disability of stroke have attracted much attention from clinicians and researchers to explore more effective and safer treatments, especially for those patients unsuitable for thrombolytic therapy and percutaneous intravascular interventions.

There are several events involved in neural cell death in brain of stroke patients [4–6]. Initially, increased apoptosis, triggered by calcium influx, impaired mitochondria, and energy depletion and followed by glutamate excitotoxicity as a result of oxygen and glucose depletion, plays a pivotal role in cell death. Then, the release of nitric oxide, oxygen free radicals, and other reactive oxygen species cause further damage to neurons. In addition, the abolishment of blood-brain barrier by the release of matrix metalloproteinases (MMPs) and other proteases from endothelial cells allows the infiltration of immune cells. Cytokines released by immune cells lead to inflammatory reaction and increased brain injury. Despite induced neurogenesis by endogeneous NSC found in several stroke models, the number and survival rate of new neurons derived from endogeneous neurogenesis are extremely low and new neurons are insufficient to replace the lost neurons in stroke victims [7].

Ischemia stroke is characterized by acute loss of neurons, astroglia, and oligodendroglia and disruption of synaptic architecture due to cerebral artery occlusion. Endogeneous cell replacement is not enough to repair adult central nervous system (CNS) in patients with stroke because of the limited renewal ability and slow turnover of neural cells. Stem cell therapy has emerged as a novel and promising candidate approach for the treatment of stroke, probably by neuroprotection and neurorepairment via secreting various neural trophic factors and replacing damaged neurons. Most of basic and translational researches are focus on three types of stem cells, including embryonic stem cell (ESCs), neural stem cell (NSCs), and mesenchymal stem cell (MSCs).

## 2. Embryonic Stem Cells (ESCs)

ESCs, derived from the inner cell mass of preimplantation embryo, possess the ability of unlimited self-renew and potential of differentiation into virtually any cell types of the organism. The advantage of ESCs is based on its capability of unlimited expansion in vitro to meet the needed amount of cells. In addition, ESCs can be induced to differentiate into neural lineage under specific culturing condition in vitro [8–12]. Hence, ESCs has been initially considered as an ideal source of transplanted cell for the treatment of neural disorders. After transplantation of mouse ESCs into rat cortex with a severe focal ischemia, ESC-derived cells expressing cell surface markers of neurons, astrocytes, oligodendrocytes, and endothelial cells could be found in the lesion cavity, and improved structural repair and functional recovery has been demonstrated [13]. Intrastriatal transplantation of mouse ESCs or ESC-derived neuron-like cells improved the dopaminergic function and subsequently recovered behavioral dysfunction in focal ischemic rats subjected to middle cerebral artery occlusion (MCAO) [14]. Intracerebral transplantation of mouse ESCs could improve the motor and sensory function of rat with MCAO and reduce the infarct size [15]. The disadvantages of ESC are its malignant transformation and teratoma formation [16–18]. Ethical concerning, limited sources, and related high incidence of malignant transformation restrict the wide application of ESCs. Hence, the studies about ESCs application in the treatment of stroke were very limited.

Transplantation of differentiated cells derived from ESCs provides a promising way to avoid malignant transformation of ESCs when infused in vivo. The neural derivatives of ESCs represent potential therapeutic cells for stroke. Many studies have explored the effect of ESC-derived neural stem/progenitor cells (NSPC) in animal models of stroke [19–23]. Most results showed improved behavioral deficit, reduced infarct area, and increased differentiation into neurons after cell transplantation, despite different transplanted cell sources, different stroke animal models, and different infusion routes. However, several studies found that the grafted human ESC-derived neural cells also have the risk of teratomas formation [24, 25]. Culturing condition might reduce tumorigenesis risk of transplanted ESC-derived neural cells. For example, neural cells derived from human ESCs under defined inductive culturing condition (named SD56) did not show chromosome abnormalities after differentiation and tumor formation after implantation into ischemic rat brains and naive nude rat brains and flanks [19]. Malignant transformation of ESC-derived neural cells has been demonstrated to be related to postischemic environment probably by the stimulation of various local cytokine [26].

It is widely acknowledged that higher cerebral blood vessel density results in less possibility and later occurrence of patients suffering from stroke. Any therapeutic measure aimed at promoting angiogenesis would play a pivotal role in function recovery of stroke patients. Intra-arterial transplantation of human ESC-derived endothelial cells and mural cells significantly increased cerebral blood vessel and vascular density in the ischemic striatum, followed by reduction of the infarct volume and of apoptosis as well as acceleration of neurological recovery in mice with transient MCAO [27].

## 3. Inducible Pluripotent Stem Cells (iPSCs)

iPSCs, reprogrammed from somatic cells with defined factors, become more attractive in the field of regenerative medicine. iPSCs has been initially induced from mouse embryonic or adult fibroblasts by introducing four factors, Oct3/4, Sox2, c-Myc, and Klf4 [28]. These cells exhibit the morphology and growth properties of ESCs and express ESC marker genes. In vivo subcutaneous transplantation of iPSCs into nude mice results in tumors containing a variety of tissues from all three germ layers. Later, iPSC has also been generated from various cell types including umbilical cord, placental mesenchymal stromal cells, neural stem cells, and adipose-derived precursor cells using the same technique [29, 30]. The benefit of iPSCs is the proliferative capacity and multipotential differentiation. In contrast to ESCs, ethical problem can be obviated in iPSC application. Chen et al. investigated the therapeutic effects of subdural transplantation of iPSCs mixed with fibrin glue on cerebral ischemic rats induced by MCAO [31]. It was shown that subdural transplantation of iPSCs can effectively reduce the total infarct volume and greatly improve the behavior of rats with MCAO to perform rotarod and grasping tasks. Attenuation of inflammation response in ischemic brain may be involved in the beneficial and protective effect of iPSCs. In another study, transplantated iPSCs from adult human fibroblasts in rat MCAO model can migrate to the injured brain area, and sensorimotor function has been significantly improved [32]. However, in one study, transplantation of iPSCs into mouse brain of transient MCAO did not lead to behavioral improvement [33]. Notably, it was found that a part of iPSCs differentiated into neuroblasts and neurons, indicating a promising therapeutic approach to supply sufficient neuronal cells for ischemia stroke. One concern with regard to iPSC application is the cellular immunogenicity. It was found that teratomas formed by iPSCs from B6 mouse embryonic fibroblasts were mostly immune-rejected by B6 recipients [34]. In other words, iPSCs, even autologous, might provoke immune rejection. Considering transcription factors of known oncogenicity used in reprogramming process, tumorigenesis property becomes another concern with iPSCs. In fact, iPSCs has been shown to form teratoma after transplanting to the ischemic brain of mice [33, 35]. iPSCs have promising potential to treat ischemic stroke if tumorigenesis is properly controlled.

## 4. Neural Stem Cells (NSCs)

### 4.1. Endogenous NSCs

Adult NSCs are mainly located in the dentate gyrus of the hippocampus, the subventricular zone (SVZ), and the olfactory bulb [36]. Many experimental studies have shown an increased proliferation of NSCs within the SVZ in animal model of MCAO, which persisted at least for four months after ischemia and probably triggered by a series of cytokines and chemokines such as SDF-1, VEGF, monocyte chemoattractant protein (MCP)-1, and macrophage inflammatory protein (MIP)-1 [37–41]. Several signaling pathways were also involved in stroke-induced neurogenesis including Notch, retinoid, bone morphogenetic protein (BMP), tumor necrosis factor-alpha (TNF-*α*), and sonic hedgehog [42–46]. Endogenous NSCs function locally by producing neurotrophic factors such as NGF and glial cell line-derived neurotrophic factor (GDNF); regulating the inflammatory environment; producing proangiogenic complexes including netrin-4, laminin, and integrins; and secreting factors promoting synaptic plasticity such as thrombospondins [47, 48]. However, the number and survival rate of neuron from these proliferative cells were extremely low, probably because of changed surrounding environment with high concentration of inflammatory cytokines. Various approaches aimed at promoting endogenous neurogenesis by enhancing endogenous NSC proliferation, survival, and differentiation have provided a promising way for the treatment of stroke. For example, increased concentration of some cytokines such as brain derived neurotrophic factor (BDNF) and VEGF in local area by gene modification or direct injection drastically promote the migration of endogeneous NSPC to injured brain areas [49, 50]. Erythropoietin (EPO) can enhance the neurogenesis and angiogenesis via BDNF and VEGF in rat stroke model [51]. Sphingosine 1-phosphate (S1P) significantly increased endogenous NPC migration toward the injured central nervous system. S1P2R antagonist upregulates the migration responses induced by S1P, and augments endogenous NPC migration toward the ischemic insult [52]. Recently, minocycline has also been demonstrated to enhance the endogenous NSC activity measured by [^18^F]FLT PET in both the SVZ as well as the hippocampus after systemic administration into rats subjected to permanent cerebral ischemia [53]. However, in a large clinical trial, overall death rate of ischemic stroke patients treated with EPO was significantly higher than placebo controls [54]. More and strict preclinical studies are necessary to ensure the safety of biological factors used for promoting endoneurogenesis.

### 4.2. Exogenous NSCs

Exogenous NSCs could be obtained from ESCs, iPSCs, bone marrow and adipose-derived MSCs, embryonic NSCs, and fetal and adult nervous systems [55]. These cells could proliferate in vitro when stimulated by various growth factors such as EGF, FGF, and leukemia-inhibiting factor (LIF) and differentiate into neurons, astrocytes, and oligodendrocytes when induced by different factors such retinoic acid. These characteristics of NSCs make them promising candidates for replacement of the lost neural cells in neurodegenerative disorders including stroke. Human fetal NSCs are less tumorigenic than embryonic stem cells, and in a clinical trial using human fetal NSCs in Batten disease, no tumors were detected in five patients 2 years after transplantation [56]. Besides, NSCs express no or low levels of MHC molecules, which obviate the problem of immunorejection [57]. However, there were also experiments showing that the expression of MHC molecules on NSCs was increased under inflammatory conditions [58, 59]. Recently, intracerebrally transplanted HUCB-NSC 3 days after ischemia completely disappeared in immunocompetent rat brain tissue at two weeks, probably resulting from innate immune response [60]. The disadvantage of NSCs includes limited capability of expansion and differentiation when cultured in vitro.

In rat model of ischemia stroke, the number of surviving embryonic NSCs was significantly higher than that of adult NSCs, accounting for its more efficient function in reducing lesion volume and improving neuronal function. More prominent Iba-1-positive inflammatory cells of rats receiving adult NSCs might be a rationale for the differences [61]. Indeed, fetal NSCs have stronger capacity of proliferation in vitro and differentiation into neurons in vivo.

Mounting experiments confirmed that transplantation of NSCs derived from various origins via different routes reduced the infarcted area and promoted the neurological function recovery in animal models with ischemic stroke (Table 1), although individual contradictory results also exist. For example, intracerebral injection of NSCs derived from human iPSCs did not reduce the infarct volume and improve functional recovery in rat ischemic stroke model, although NSC could survive and differentiate into neurons [62]. The time, dose, and type of transplanted cell might account for the different results. NSCs in combination with gene modification could enhance their survival, proliferation and migration abilities, and secrete neurotrophic factors if as a gene herapy vehicle. Over expression of some neuroprotective cytokines including VEGF, neurotrophin-3, BDNF, FGF-2, and GDNF has been reported to potentiate the therapeutic effects of transplanted NSCs in stroke animals [63–67]. Gene modification with HIF-1a also could potentiate the beneficial effect of NSCs on neurological recovery following cerebral ischemia, probably through promoting angiogenesis [68].

#### 4.2.1. Transplantation Route

Stem cells in circulation can migrate into injured brain area probably in response to chemoattractant stimuli through rolling on and adhering to endothelium and endothelial transmigration. VCAM-1 and the integrins *α*2, *α*6, and *β*1 may be involved in the communication between stem cells and endothelium. Then, NSCs are guided into targeted brain area via chemokines such as SDF-1, MCP-1 Ang-1, and Slit [81–87]. The CCL2/CCR2 interaction has recently been demonstrated to be critical for transendothelial recruitment of intravascularly delivered NSCs in response to ischemic injury [88]. A recent study revealed that the advantageous effect of intracerebral transplantation of NSC in stroke depend on high intracerebral numbers of grafted cells; however, systemic NSC delivery initiates sustained neuroprotection despite low intracerebral numbers of grafted cells via different mechanisms, like stabilization of the BBB and reduction of ROS during early reperfusion [89]. So, compared to intracerebral transplantation, intravascular delivery of NSCs obtains better distribution into the injured brain areas and avoids the process of invasive surgery.

#### 4.2.2. Time and Dose of Cell Transplantation

In a study of rat MCAO model [90], focal transplantation of human NSCs early after stroke (48 hours) resulted in better cell survival than did transplantation 6 weeks after stroke, but the delayed transplantation did not influence the magnitude of migration, neuronal differentiation, and cell proliferation in the grafts. Transplanting greater numbers of NSCs did not result in a greater number of surviving cells or increased neuronal differentiation. Optimal time and dose of cell transplantation change depending on different animal model, cell source, and infusion route. The results of various relative researches are difficult to compare. It was concluded that transplantation before maximal activation of microglia was more beneficial for cell survival.

#### 4.2.3. Mechanisms

The mechanisms underlying the improved functional recovery of ischemic stroke animal model subjected to NSCs therapy remain unclear. Cell replacement was initially recognized as the main mechanism of advantageous effect of transplanted NSCs. In most animal studies, NSCs were found to differentiate into neuronal and/or glial phenotypes [70, 91–94]. Synaptogenesis and functional electrophysiological integration of exogenous NSC into the neuronal circuitry of the host brain has also been demonstrated [95]. However, improved functional recovery could be observed during the time periods while neuronal differentiation has not been achieved. It suggests that cell replacement may not be prerequisite for the effect of stem cell on the neuronal recovery.

*Neuroprotective Effects. *The number of NSCs surviving in the lesion area was too scarce to replace lost neurons. Hence, it was supposed that neuroprotective cytokines secreted by exogenous NSCs, host stem cells, and/or other cells, such as VEGF, BDNF, NGF, and neurotrophins, might play pivotal roles in functional recovery after ischemic stroke directly or indirectly via angiogenesis, immunomodulation, endogenous neurogenesis, and so forth [96–98]. Increased dendritic plasticity and axonal rewriting in stroke have been linked with paracrine effects of NSCs possibly via VEGF and thrombospondins 1 and 2 [99].

*Endogenous Neurogenesis*. Injection of NSCs into rat cortical infarct cavity has been found to stimulate neurogenesis in the SVZ ipsilateral to stroke, as demonstrated by increased numbers of cells expressing the early neuronal lineage marker Dcx 60 d posttransplant [100]. Transplantation of human embryonic NSCs into cortical peri-infarction in rat 24 h after ischemia increased the number of BrdU^+^ cell in SVZ [80]. Although the experiment did not directly answer whether enhanced neurogenesis contribute to the neurological function improvement, ablation of endogenous neurogenesis in transgenic mice expressing *Herpes simplex virus* thymidine kinase under control of the Dcx promoter increases infarct size and exacerbates postischemic sensorimotor behavioral deficits in another study [101]. 

*Modulation of Inflammatory and Immune Response*. Inflammatory and immune response mediate secondary injury after acute ischemia insult. Microglia is the main inflammatory regulators in focal brain tissue. It was shown that intravenous administration of NSCs reduced OX-42^+^ microglia and MPO^+^ neutrophil infiltration into brain lesion and also attenuated both cerebral and splenic activations of TNF-a, IL-6, and NF-kB, which promoted neuroprotection in rat stroke model [102]. Some inflammatory regulators, such as TNF-a, IL-1*β*, IL-6, and leptin receptors, have also been downregulated in ischemia brain after transplantation of NPCs [77]. Human immortalized neural stem cell lines (CTX0E03 cell line) has been demonstrated to significantly increase the proliferative DCX^+^ neuroblast in rat MCAO model. The author inferred that concomitantly increased proliferating microglia might mediate this proproliferation effect of CTX0E03 cells [103]. Upregulation of class MHC-I in brain lesion, crucial to neuronal development, differentiation, synaptic plasticity, and behavior, has also been linked with improved neurological function in ischemia rat following NSC transplantation [76].

*Angiogenesis*. Angiogenesis is important for formation of new brain microvessels and functional recovery after ischemic stroke. Enhanced angiogenesis has been associated with functional recovery after NSCs transplantation following stroke [47, 80, 104, 105]. For example, angiogenesis, as indicated by BrdU and vWF staining in cortical peri-infarct regions, has been strengthened by grafted human embryonic NSCs in rat stroke model at 7 and 14 days [80].

## 5. Mesenchymal Stem Cells (MSCs)

MSCs constitute a population of nonhematopoietic cells in the bone marrow from which these were identified for the first time [106]. Subsequently, MSCs have been successfully isolated from almost all tissues in mammals including circulating blood, UCB, menstrual blood, placenta, heart, adipose tissue, skeletal muscle, pancreas, and dental pulp [107]. Three criteria have been proposed to identify MSCs by the Mesenchymal and Tissue Stem Cell Committee of the International Society for Cellular Therapy, including the plastic adherence of the isolated cells in culture, the expression of CD105, CD73, and CD90 in more than 95% of the culture, and their lack of expression of markers including CD34, CD45, and CD14 or CD11b, CD79a or CD19, and HLA-DR in more than 95% of the culture and the differentiation of the MSCs into osteoblasts, adipocytes, and chondroblasts in vitro [108]. MSCs are also capable of differentiating into neural cells, hepatocytes, insulin-producing cells (IPCs), and so forth [109].

The ability of self-renewal and differentiation into neural cells in vitro, as demonstrated by expression of neuronal markers such as NeuN, migration towards sources of lesions in the brain, and non-ethical and tissue-rejection related concerns make MSCs a promising therapeutic approach in stroke treatment. In preclinical experimental studies, transplantation of human or rat MSCs displayed significant effect of functional improvement in animal stroke (Table 2). Gene modification with exogeneous cytokines such as GDNF, BDNF, FGF-2, placental growth factor (PlGF), and angiopoietin-1 fortified the effective roles of MSCs [110–114].

### 5.1. Transplantation Route

Intravenously or intracerebrally transplanted MSCs could migrate into the injured brain and promote functional improvement in experimental animal models of stroke, although sometimes reduced infarct volume was not observed. The capability of migration of MSCs may be mediated by increased chemokines such as SDF-1 in surrounding environment and CXCR4 presented on MSCs [141]. There are also many other potential factors, such as IL-8, MCP-1, MIP-1a, and VEGF, that might be involved in the migration of MSCs into injured brain [142, 143]. Intravenous delivery of MSCs is superior to intracerebral injection because it is less invasive, more extensively neuroprotective, and more easily utilized in clinic.

### 5.2. Time and Dose of Cell Transplantation

The optimal dose of cell therapy currently remains unclearly defined. The dose of 1-2 million cells/kg of body weight was suggested for clinical study [144]. The optimal time of transplantation depends on the dynamically changed environment in injured brain. Early delivery of MSCs probably plays neuroprotective roles because of its counteract against increased toxicity and inflammatory response. Transplantation of cells at 2-3 weeks after ischemia probably is more superior in enhancing endogenous neuronal repair such as plasticity, angiogenesis, and neurogenesis, which are more intense at that time. Enhanced functional recovery was observed even at 1 year after administration in ischemic rats [145].

### 5.3. Mechanisms

Several mechanisms have been explored to account for the beneficial effect of MSCs on experimental stroke model (Table 2). Although MSCs has been demonstrated to be capable of differentiating into cells of neural lineage in vitro and express neuronal or glial markers in ischemic brain of animal models [110, 115, 126, 136, 146–148], the survival number of grafted and differentiated cells only took a small portion. Moreover, some studies explore the function of neuronal cells from MSCs with much controversy [3, 149–152]. Hence, cell replacement might not be mainly responsible for the beneficial effect of MSCs on ischemic brain injury in vivo. MSCs might exert its effects via a series of secreted trophic factors which directly or indirectly promote ischemic brain tissue repair. MSCs is stimulated to secrete various neurotrophic factors including cytokines, chemokines, and extracellular matrix protein by damaged surrounding environment. Secretion of trophic factors by MSCs and/or MSC-stimulated resident cerebral cells has been considered to contribute to the beneficial effects mentioned above. MSCs constitutively express BDNF, which was significantly increased when MSCs was transplanted into MCAO model. MSCs overexpressing BDNF showed stronger therapeutic effects than original MSCs alone [110]. Other neurotrophic factors, such as HGF, VEGF, NGF, bFGF, FGF-2, and IGF-1, have been demonstrated to be implicated in endogenous repair mechanisms mediated by MSCs [126, 134, 137, 153, 154]. The trophic factors might play critical roles in neuroprotection, angiogenesis, synaptogenesis, endogenous neurogenesis, and inflammatory and immune response.

#### 5.3.1. Neuroprotective Effects

Several studies found that numerous neurotrophic factors such as SDF-1, VEGF, GDNF, BDNF, NGF, IGF, EGF, and bFGF was significantly increased in ischemic animal brain after MSCs treatment [115, 119–121, 125, 128, 133, 134]. These increased neurotrophic factors were secreted by MSCs directly and/or stimulated host cells indirectly. The neuroprotection mediated by these neurotrophic factors including antiapoptosis, increasing neuron survival, antioxidation, antiglutamate excitotoxicity, and anti-inflammatory activity probably account for beneficial effects of MSCs on ischemic brain injury. Recently, MSCs could increase tPA activation and downregulate PAI-1 levels in the ischemic boundary zone, which promote the axonal and synaptophysin production and finally improve functional recovery in rat model of stroke [117, 118].

#### 5.3.2. Angiogenesis

Moreover, MSCs have also been described to favour angiogenesis and synaptogenesis [115, 119, 122–125, 129, 131, 140, 155–157]. Increased expression of *β*1-integrin, the modulatory effect of macrophage/microglial cells, and enhancement of neurotrophic factor secretion, mediated by MSCs, might contribute to the induction of new vessels [115]. Other trophic factors secreted by MSCs which probably contributed to enhanced angiogenesis include VEGF, BDNF, IGF-1, bFGF, GDNF, and TGF [135, 158, 159]. In addition, it was found that the number of endothelial progenitor cells markered by CXCR4 was significantly increased in brain of adipose-derived MSC (Ad-MSC) treated ischemic rats. Additionally, Ad-MSC could differentiate into endothelial cell phenotype as demonstrated by vWF staining [119]. These results indicate that differentiation into endothelial cell and/or mobilization of endothelial progenitor cells might be involved in proangiogenic effect of MSCs in stroke. Recently, it was shown that Notch signalling pathway was involved in MSC-induced angiogenesis in ischemic brain [160].

#### 5.3.3. Endogenous Neurogenesis

Notably, increased endogenous neurogenesis might be another mechanism by which MSCs improve the neurological function in ischemic stroke [119, 125, 127]. When human umbilical cord-derived MSCs were implanted into rats two weeks after MCAO, nestin-positive endogenous stem cells in the hippocampus were significantly increased at 35 days [127]. Intravenous infusion of AdMSCs significantly increased expression of doublecortin in infracted brain area which is an indication of migrating neuroblast [119]. In another study, enhanced proliferation, migration, and differentiation of endogenous neural stem/progenitor cells has also been demonstrated in the SVZ and subgranular zone of the hippocampus when Flk-1^+^ hBMSCs was intracerebrally injected into ischemic brain in rats [125]. It is speculated that enhanced endogenous neurogenesis might be attributed to increased angiogenesis and subsequent improved CBF. Moreover, the influence of the trophic factor cannot be excluded; for example, BDNF can stimulate neurogenesis directly [161].

#### 5.3.4. Modulation of Inflammatory and Immune Response

Inflammatory and immune response modulation by MSCs is a mechanism underlying neuronal protection in ischemic stroke. After Ad-MSCs were intravenously injected into rat model of stroke, mRNA expressions of IL-18, TLR-4, and plasminogen activator inhibitor (PAI)-1 in infarcted brain area, indexes of inflammation, were significantly reduced [119]. hBMSC has been shown to upregulate IL-10 expression in a non-human primate ischemia model, which probably accounts for attenuation of astroglial reactivity, antiapoptotic effect, and neurogenesis [128]. MSCs administration into MCAO rats could cause amplification of activated CD11+ microglia and reactive GFAP+ astrocytes in the peri-infarct area far greater and more long lasting than that seen after stroke alone. Although expression of blood cytokines/chemokines (IL-13, MMP2, and MIP) and growth factors/receptors (VEGF, neuropilin, EPOR, TROY, NGFR, and RAGE) were upregulated following MSCs administration, the causal roles that these blood-borne factors play in improving brain structure and function after MSCs injection in the MCAO rat remain to be validated [129]. However, in one study, systemic inflammatory cytokine levels (IL-6, TNF-*α*, interferon-*γ*, and MCP-1) remained unchanged in the sera of mice after cerebral ischemia and MSCs transplantation [162]. Recently, MSCs has been demonstrated to decrease MCP-1 expression and subsequent infiltration of CD68+ cells in ischemic brain, whereas transplantation of TGF-*β*1-silenced MSCs cannot exert similar effects. Hence, it is reasonable that MSCs secrete TGF-*β*1 to suppress immune propagation in the ischemic rat brain [163].

### 5.4. Clinical Trials

12 patients with ischaemic grey matter, white matter, and mixed lesions were included in a nonrandomized, open-label trial. Autologous MSCs, expanded in autologous serum, were delivered intravenously 36–133 days after stroke. There were no cell infusion related side effects such as tumours, abnormal cell growths, neurological deterioration, or venous thromboembolism. As a result, the median daily rate of NIHSS change increased during the first week after infusion, and mean lesion volume as assessed by MRI was reduced by >20% at 1 week after cell infusion. Although this nonblinded study did not exclude placebo effects or natural recovery of stroke, it provided evidence that transplantation of autologous MSCs is feasible and safe [164].

In a clinical trial, four patients with stroke (three with ischemic and one with hemorrhagic stroke) in the middle cerebral artery territory were recruited. One single dose of 2 × 10^7^ UCMSCs was infused into the MCA. No side effects such as stroke, death, fever, and rash were observed during the 6-month followup. Improved modified Rankin scale was observed in two of the ischemic patients. However, the efficacy and safety of the approach cannot be determined due to small number of enrolled patients and lack of control [165].

In a recent study, 50–60 million bone marrow-derived MSCs were infused intravenously into patients with diagnosed stroke from 3 months to 2 years of index event. There was no mortality or cell related adverse reactions in stem cell-treated patients. Modified Barthel Index (mBI) showed statistical significant improvement in the stem cell group. An increased neural plasticity was observed after stem cell infusion indicating neural plasticity. The authors concluded that stem cells act as “scaffolds” for neural transplantation and may aid in repair mechanisms in stroke [144].

## 6. Current Concerns

### 6.1. Cell Homing

Intravenous injection of cells in rats after cerebral ischemia resulted in high accumulation of cells into internal organs such as lungs, liver, and spleen [166]. Intra-arterial infusion was usually accompanied by high incidence of microocclusion, although it could circumvent the filtering organs [167]. Approaches to improve cell homing and efficiency of cell therapy include cell sorting, altered culture conditions, and cell surface modifications. Enrichment of NSCs by FACS for the surface integrin CD49d has been demonstrated to promote cell homing to the area of stroke in mice and improve behavioral recovery [47]. Cell surface engineering may also target cells to tissue of interest. Cells treated with a proteolytic enzyme (pronase) could transiently modify cell surface adhesion proteins [168]. Priming with valproate and/or lithium could promote the homing and migration ability of MSCs in a rat MCAO model, which was likely mediated by VPA-induced CXC chemokine receptor 4 overexpression and lithium-induced matrix metalloproteinase-9 upregulation [138].

### 6.2. Cell Survival

Several factors may affect cell survival in the acute phase of cerebral infarction, including limited blood supply, hypoxia, trophic factor deficiency, oxidative stress, inflammatory response, and others [169]. Gene modification with various factors such as Bcl-2 and PlGF significantly promoted the survival of ESCs and MSCs [13, 111]. Overexpression of growth factor genes including VEGF, GDNF, BDNF, and Akt1 was able to significantly promote the survival of NSCs in stroke animal model [67, 102, 170, 171]. Preconditioning with IL-6 protected the grafted NSCs from ischaemic reperfusion injury in a mouse ischemic stroke model [172]. The survival, proliferative capacity, and paracrine effects of NSCs were enhanced by minocycline preconditioning when intracerebrally transplanted in stroke brain accounting for the improved neurological recovery compared with nonconditioned NSCs. The applications of biomaterial scaffolding also have the potential to enhance the survival of NSCs when intracerebrally transplanted in ischemic brain [74]. Recently, transduction of TAT-heat shock protein 70 (Hsp70) in vitro, which reduces apoptosis and inflammation after hypoxic-ischemic injury, significantly boost the survival of NSCs intracerebrally transplanted in poststroke mice brain [89]. It seemed that hypoxia-treated BMSCs survive better, more BMSCs home to ischemic region, and exhibit a superior property of promoting angiogenesis and neurogenesis after administration into rat subjected to MCAO [136].

### 6.3. Cell Tracking

In vivo cell tracking also remained an unresolved critical issue. Various cell labeling technique, for example, fluorescent proteins, iron oxide, iron containing agents (e.g., Feridex), magnetodendrimers, particles conjugated with Tat peptides, and paramagnetic particles (gadolinium-diethylene triamine penta-acetic acid [Gd-DTPA]) [173–175], make it possible to track cell fate and migration in combination with MRI after transplantation. There are advantages and shortcomings with regard to labeling efficiency, toxic effects, manufacturing and sensitivity. For example, simple cell endocytosis and lipofectamine-mediated methods of transfection have low labeling efficiencies [176]. It has been demonstrated that supraparamagnetic substances, such as iron oxide, was harmful to cell signaling and function [177]. Magnetodendrimers and particles conjugated with Tat peptides require complicated methods of manufacture. SPECT imaging allows whole body biodistribution studies based on cell labeling with ^111^In-oxine. Besides, this technique also has advantage of high sensitivity, short scanning times, and repeated scanning over several days. The disadvantage mainly include toxicity, and tested signal may come from cell debris rather than surviving cells [178].

### 6.4. Safety

Safety represents a critical concern before stem cells are allowed to be extensively used in clinic. Recently, a meta-analysis of clinical trials including MEDLINE, EMBASE, and the Cochrane Central Register of Controlled Trials (to June 2011) did not detect associations between MSCs treatment and the development of acute infusional toxicity, organ system complications, infection, death, or malignancy. There was, however, a significant association between MSCs administration and transient fever [179]. Although current clinical trials indicate stem cell therapy for stroke is feasible and safe, robust scientific data is deficient.

### 6.5. Gene Therapy

Stem cell-based gene therapy represents a novel potential therapeutic strategy for ischemic stroke in future. Stem cells per se can secret various neurotrophic factors besides as gene delivery vehicles. Transplantation of gene modified-stem cells overexpressing diverse neurotrophic factors such as VEGF, BDNF, GDNF, PIGF, ANG-1, HGF, NGF, EPO, and noggin has been demonstrated to significantly improve the functional recovery in stroke compared to stem cells only [13, 111, 122, 133, 170, 180–183]. Overexpression of NGF and noggin in BMSCs could upregulate the presence in ischemic brain and neuronal differentiation of BMSCs.

## 7. Conclusion

**Figure 1:**
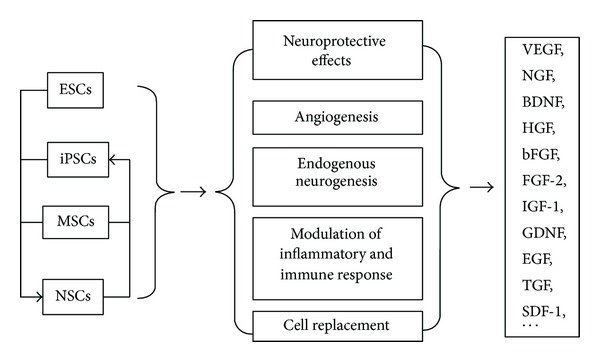
A hierarchy sketch of stem cells applicable in stroke and the mechanisms of action in the neuroregeneration/neuroprotection.

Conclusively, stem cells have become attractive candidates for cell therapy in stroke treatment of which so far no ideal therapeutic measures are available. The beneficial effects of stem cells might include neuroprotection, angiogenesis, inflammatory, and immune response (Figure 1). Although most animal studies demonstrated that impaired neural function has been significantly improved after administration of various stem cells, many critical issues have to be addressed before clinical application. For example, optimal cell source, dosing, timing and routing, adverse event monitoring, and management need to be urgently determined. Better understanding of the mechanisms of stem cells in treating stroke will help resolve issues above. Large clinical trials are also necessary. In future, stem cell combined with gene therapy will play important roles in experimental and clinical application.

## Figures and Tables

**Table 1:** Summary of NSC transplantation experiments in ischemic stroke models.

Model	Cell	Route	Time	Behavioral measures	Outcome	Mechanism
rMCAO [69]	fetal hNSC	IC	7 d	NA	NA	Neuronal differentiation

rMCAO [70]	hNSC	IV	1 d	TIA, MLPT, rotarod test	IFR	Migration and differentiation marker for neurons or astrocytes

rMCAO [71]	hNSC	IV	24 h	MLPT rotarod test	IFR	↑EBA immunoreactivities

rMCAO [19]	hESC-derived NSC	IC	7 d	cylinder test	IFR	NA

pMCAO [72]	hNSC	LT	24 h	mNSS	IFR no reduced infarct size	↑Bcl-2

rMCAO [73]	hNSC	IC	4 w	Bilateral asymmetry test (BAT), rotameter test	IFR.	Paracrine trophic endothelial differentiation ↑Neurogenesis

rMCAO [74]	hNPC	LT	3 w	Cylinder test, adhesive-removal test	IFR (sensorimotor/ cognitive functions)	↓Neuronal differentiation

PI in cortex [75]	mNSC	IV	24 h	Adhesive-removal test	IFR (sensorimotor function)	↑Dendritic plasticity ↑Activated microglial cells ↓Endogenous neurogenesis

rMCAO [61]	Embryonic NSC	IC	1 h	Limb placement test, rotarod test, cylinder test	Reduced infarct volume IFR	Cell replacement Trophic effects

mMCAO [47]	CD49d ^+^NSCs	Intracarotid	48 h	Rotarod	IFR	↑Cell homing

rMCAO [76]	NSC	IC*	7 d	Somatosensory response, trap channel test	IFR	↑MHC-I

mMCAO [77]	NPC	IV	72 h	mNSS	IFR	Anti-inflammatory effect

rICH [78]	hNSC	IV	24 h	Rotarod test, modified limb placing tests	IFR	Differentiation into neuron and astrocytes

rMCAO [79]	rNSC and collagen I	IC	24 h	mNSS	IFR	Synapse formation

rMCAO [80]	embryonic hNSC	IC	24 h	NA	NA	↑Endogenous cell proliferation in SVZ, angiogenesis in peri-infarct zone

NA: not assessed; h: human; IC: intracerebral; IV: intravenous; IA: intra-arterial; LT: local transplantation; EBA: endothelial barrier antigen; IFR: improved functional recovery; TIA: turning in an alley test; MLPT: modified limb placing test; PI: photothrombotic ischemia.

**Table 2:** Summary of MSC transplantation experiments in ischemic stroke models.

Model	Cell	Route	Time	Behavioral measures	Outcome	Mechanism
rMCAO [115]	hUCMSC	IC	One week	Elevated body swing test, locomotor activity	IFR ↑neuronal activity	Neural differentiation into neuron, glia, EC ↑Neurotrophic factors (SDF-1, GDNF, BDNF) ↑Angiogenesis

rMCAO [116]	BMSC	ICA	24 h	Rotarod test adhesive-removal test mNSS	IFR	Production of trophic factors

mMCAO [110]	BDNF-modified hBMSC	IC	24 h	Limb placement test, treadmill test	IFR	↓Apoptosis; Differentiation (NeuN, GFAP)

mMCAO [117]	mBMSC	IV	1 d	NA	NA	↑tPA ↓PAI-1

mMCAO [118]	mBMSC	IV	1 d	mNSS Foot-fault tests	IFR; ↓lesion volume	↑tPA level

rMCAO [119]	Ad MSC	IV	0, 12 and 24 h	Corner test	↓infarct area IFR (sensorimotor)	↑Angiogenesis, neurogenesis, homing ↓Apoptosis, inflammation, and oxidation

rMCAO [120]	hBMSC	IV	1 d	mNSS	IFR	↑Growth factors; ↓apoptosis proliferated endogenous cells

rMCAO [121]	hBMSC	IV	12 h	Morris water maze, treadmill test	IFR ↓lesion volume	Neuroprotective effect

rMCAO [122]	Ang-1 modified hBMSC	IV	6 h	Treadmill stress test	IFR ↓gross lesion volume	↑Angiogenesis

rMCAO [123]	pMSC	IV	6 h	Treadmill stress test	IFR ↓lesion volume	↑Induced angiogenesis

rMCAO [124]	hBMSC	IV	6 h or 6, 24, 48 h	Treadmill stress test	IFR ↓lesion volume	↑Capillary vessels

rMCAO [125]	Human BMSC	IC	3 d	mNSS adhesive-removal somatosensory test	IFR, ↓infarct volume and↑glucose metabolism	↑Angiogenesis ↑Endogenous neurogenesis ↓Apoptosis

rMCAO [126]	MSC	IA	1 d	Adhesive-removal, mNSS	IFR	Differentiation into astrocytes and neurons

rMCAO [127]	hUC-MSC	IC	14 d	Neurobehavioral assessment neurologic deficit score	IFR ↓infarct volume	Neuroprotective effects ↑Endoneurogenesis

ape ischemia model [128]	hBMSC	IC	7 d	mNSS	IFR ↓ischemia area	↑IL-10, neurogenesis ↓Astroglial reactivity and apoptosis

rMCAO [129]	rBMSC or hBMSC	IV	1 d or 7 d	mNSS	IFR	↑Activated CD11+ microglia, reactive GFAP+ astrocytes, and blood vessel formation modified blood levels of specific cytokines/chemokines and growth factors/receptors

rMCAO [130]	rBMSC	ICA	2 h	NA	↓lesion size	Activated microglia

rMCAO [131]	rBMSC	IV	on the day of MCAO and three days later	Morris water maze	IFR Reduction of the infarct volumes	↑Angiogenesis and subventricular zone cells proliferation

mTCCO [132]	hBMSC	ICA	1 d	Open-field behavior test	IFR	↓Neuronal death and modulation of inflammatory and immune responses

rMCAO [133]	Gene-transferred MSCs	IC	2 h or 24 h	mNSS	IFR ↓infarction areas	Neuroprotective effect

rMCAO [134]	hBMSC	IV	1 d	mNSS	IFR	↑IGF-1, IGF-1R

rMCAO [135]	BMSC	IV	24 h	mNSS foot-fault tests	IFR no difference in ischemic volume	↑trophic factor

rMCAO [136]	hypoxic preconditioned or normal BMSC	IV	24 h	Rotarod test	IFR	↓Microglia activity ↑Differentiation into neuronal and vascular endothelial cells

rMCAO [137]	B10 line of hMSC	IV	24 h	mNSS score	IFR ↓infarct volume	Providing IGF-1 inducing VEGF, EGF, and bFGF in host brain

rMCAO [138]	Valproate/ lithium-primed MSC	IV	24 h	Rotarod test, mNSS body asymmetry test	IFR ↓infarct volume ↑angiogenesis	↑CXCR 4, MMP-9

rMCAO [139]	pMSC	IV	8 h 8 h and 24 h	Beam walk test mNSS	IFR ↓lesion volume	Immunomodulation soluble factor secretion ↑Astroglial reactivity

rMCAO [140]	BM-MSC Ad-MSC	IV	30 min	Rogers and rotarod tests	IFR no reduction in infarct volume	↑VEGF, SYP, Olig2, NF; decreased GFAP

h: human; p: human placenta; Ad: adipose-derived; m: mouse; r: rat; ICA: intracarotid arterial; IC: intracerebral; IV: intravenous; IA: intra-arterial; mNSS: modified neurological severity score; IFR: improved functional recovery; NA: not assessed.

## References

[1] Marler JR (1995). Tissue plasminogen activator for acute ischemic stroke. *New England Journal of Medicine*.

[2] Sandercock P, Berge E, Dennis M (2004). Cost-effectiveness of thrombolysis with recombinant tissue plasminogen activator for acute ischemic stroke assessed by a model based on UK NHS costs. *Stroke*.

[3] Thomalla G, Sobesky J, Köhrmann M (2007). Two tales: hemorrhagic transformation but not parenchymal hemorrhage after thrombolysis is related to severity and duration of ischemia—MRI study of acute stroke patients treated with intravenous tissue plasminogen activator within 6 hours. *Stroke*.

[4] Dirnagl U, Iadecola C, Moskowitz MA (1999). Pathobiology of ischaemic stroke: an integrated view. *Trends in Neurosciences*.

[5] Lo EH, Dalkara T, Moskowitz MA (2003). Mechanisms, challenges and opportunities in stroke. *Nature Reviews Neuroscience*.

[6] Barkho BZ, Zhao X (2011). Adult neural stem cells: response to stroke injury and potential for therapeutic applications. *Current Stem Cell Research and Therapy*.

[7] Arvidsson A, Collin T, Kirik D, Kokaia Z, Lindvall O (2002). Neuronal replacement from endogenous precursors in the adult brain after stroke. *Nature Medicine*.

[8] Bain G, Kitchens D, Yao M, Huettner JE, Gottlieb DI (1995). Embryonic stem cells express neuronal properties in vitro. *Developmental Biology*.

[9] Okabe S, Forsberg-Nilsson K, Spiro AC, Segal M, McKay RDG (1996). Development of neuronal precursor cells and functional postmitotic neurons from embryonic stem cells in vitro. *Mechanisms of Development*.

[10] Reubinoff BE, Itsykson P, Turetsky T (2001). Neural progenitors from human embryonic stem cells. *Nature Biotechnology*.

[11] Zhang S-C, Wernig M, Duncan ID, Brüstle O, Thomson JA (2001). In vitro differentiation of transplantable neural precursors from human embryonic stem cells. *Nature Biotechnology*.

[12] Ying Q-L, Stavridis M, Griffiths D, Li M, Smith A (2003). Conversion of embryonic stem cells into neuroectodermal precursors in adherent monoculture. *Nature Biotechnology*.

[13] Wei L, Cui L, Snider BJ (2005). Transplantation of embryonic stem cells overexpressing Bcl-2 promotes functional recovery after transient cerebral ischemia. *Neurobiology of Disease*.

[14] Yanagisawa D, Qi M, Kim D-H (2006). Improvement of focal ischemia-induced rat dopaminergic dysfunction by striatal transplantation of mouse embryonic stem cells. *Neuroscience Letters*.

[15] Tae-Hoon L, Yoon-Seok L Transplantation of mouse embryonic stem cell after middle cerebral artery occlusion. *Acta Cirúrgica Brasileira*.

[16] Solter D (2006). From teratocarcinomas to embryonic stem cells and beyond: a history of embryonic stem cell research. *Nature Reviews Genetics*.

[17] Reubinoff BE, Pera MF, Fong C-Y, Trounson A, Bongso A (2000). Embryonic stem cell lines from human blastocysts: somatic differentiation in vitro. *Nature Biotechnology*.

[18] Thomson JA (1998). Embryonic stem cell lines derived from human blastocysts. *Science*.

[19] Daadi MM, Maag A-L, Steinberg GK (2008). Adherent self-renewable human embryonic stem cell-derived neural stem cell line: functional engraftment in experiment stroke model. *PLoS ONE*.

[20] Kim D-Y, Park S-H, Lee S-U (2007). Effect of human embryonic stem cell-derived neuronal precursor cell transplantation into the cerebral infarct model of rat with exercise. *Neuroscience Research*.

[21] Hicks AU, Lappalainen RS, Narkilahti S (2009). Transplantation of human embryonic stem cell-derived neural precursor cells and enriched environment after cortical stroke in rats: cell survival and functional recovery. *European Journal of Neuroscience*.

[22] Bühnemann C, Scholz A, Bernreuther C (2006). Neuronal differentiation of transplanted embryonic stem cell-derived precursors in stroke lesions of adult rats. *Brain*.

[23] Hayashi J, Takagi Y, Fukuda H (2006). Primate embryonic stem cell-derived neuronal progenitors transplanted into ischemic brain. *Journal of Cerebral Blood Flow and Metabolism*.

[24] Sonntag K-C, Pruszak J, Yoshizaki T, Van Arensbergen J, Sanchez-Pernaute R, Isacson O (2007). Enhanced yield of neuroepithelial precursors and midbrain-like dopaminergic neurons from human embryonic stem cells using the bone morphogenic protein antagonist noggin. *Stem Cells*.

[25] Brederlau A, Correia AS, Anisimov SV (2006). Transplantation of human embryonic stem cell-derived cells to a rat model of Parkinson’s disease: effect of in vitro differentiation on graft survival and teratoma formation. *Stem Cells*.

[26] Seminatore C, Polentes J, Ellman D (2010). The postischemic environment differentially impacts teratoma or tumor formation after transplantation of human embryonic stem cell-derived neural progenitors. *Stroke*.

[27] Oyamada N, Itoh H, Sone M (2008). Transplantation of vascular cells derived from human embryonic stem cells contributes to vascular regeneration after stroke in mice. *Journal of Translational Medicine*.

[28] Takahashi K, Yamanaka S (2006). Induction of pluripotent stem Cells from mouse embryonic and adult fibroblast cultures by defined factors. *Cell*.

[29] Cai J, Li W, Su H (2010). Generation of human induced pluripotent stem cells from umbilical cord matrix and amniotic membrane mesenchymal cells. *Journal of Biological Chemistry*.

[30] Tat PA, Sumer H, Jones KL, Upton K, Verma PJ (2010). The efficient generation of induced pluripotent stem (iPS) cells from adult mouse adipose tissue-derived and neural stem cells. *Cell Transplantation*.

[31] Chen S-J, Chang C-M, Tsai S-K (2010). Functional improvement of focal cerebral ischemia injury by subdural transplantation of induced pluripotent stem cells with fibrin glue. *Stem Cells and Development*.

[32] Jiang M, Lv L, Ji H (2011). Induction of pluripotent stem cells transplantation therapy for ischemic stroke. *Molecular and Cellular Biochemistry*.

[33] Kawai H, Yamashita T, Ohta Y (2010). Tridermal tumorigenesis of induced pluripotent stem cells transplanted in ischemic brain. *Journal of Cerebral Blood Flow and Metabolism*.

[34] Zhao T, Zhang Z-N, Rong Z, Xu Y (2011). Immunogenicity of induced pluripotent stem cells. *Nature*.

[35] Yamashita T, Kawai H, Tian F, Ohta Y, Abe K (2011). Tumorigenic development of induced pluripotent stem cells in ischemic mouse brain. *Cell Transplantation*.

[36] Ming G-L, Song H (2011). Adult neurogenesis in the mammalian brain: significant answers and significant questions. *Neuron*.

[37] Thored P, Arvidsson A, Cacci E (2006). Persistent production of neurons from adult brain stem cells during recovery after stroke. *Stem Cells*.

[38] Kim JS (1996). Cytokines and adhesion molecules in stroke and related diseases. *Journal of the Neurological Sciences*.

[39] Zhang ZG, Zhang L, Tsang W (2002). Correlation of VEGF and angiopoietin expression with disruption of blood-brain barrier and angiogenesis after focal cerebral ischemia. *Journal of Cerebral Blood Flow and Metabolism*.

[40] Otto VI, Gloor SM, Frentzel S (2002). The production of macrophage inflammatory protein-2 induced by soluble intercellular adhesion molecule-1 in mouse astrocytes is mediated by src tyrosine kinases and p42/44 mitogen-activated protein kinase. *Journal of Neurochemistry*.

[41] Hallbergson AF, Gnatenco C, Peterson DA (2003). Neurogenesis and brain injury: managing a renewable resource for repair. *Journal of Clinical Investigation*.

[42] Wang X, Mao X, Xie L, Greenberg DA, Jin K (2009). Involvement of Notch1 signaling in neurogenesis in the subventricular zone of normal and ischemic rat brain in vivo. *Journal of Cerebral Blood Flow and Metabolism*.

[43] Chou J, Harvey BK, Chang C-F, Shen H, Morales M, Wang Y (2006). Neuroregenerative effects of BMP7 after stroke in rats. *Journal of the Neurological Sciences*.

[44] Plane JM, Whitney JT, Schallert T, Parent JM (2008). Retinoic acid and environmental enrichment alter subventricular zone and striatal neurogenesis after stroke. *Experimental Neurology*.

[45] Sims JR, Lee S-W, Topalkara K (2009). Sonic hedgehog regulates ischemia/hypoxia-induced neural progenitor proliferation. *Stroke*.

[46] Iosif RE, Ahlenius H, Ekdahl CT (2008). Suppression of stroke-induced progenitor proliferation in adult subventricular zone by tumor necrosis factor receptor 1. *Journal of Cerebral Blood Flow and Metabolism*.

[47] Guzman R, de los Angeles A, Cheshier S (2008). Intracarotid injection of fluorescence activated cell-sorted CD49d-positive neural stem cells improves targeted cell delivery and behavior after stroke in a mouse stroke model. *Stroke*.

[48] Staquicini FI, Dias-Neto E, Li J (2009). Discovery of a functional protein complex of netrin-4, laminin *γ*1 chain, and integrin *α*6*β*1 in mouse neural stem cells. *Proceedings of the National Academy of Sciences of the United States of America*.

[49] Benraiss A, Chmielnicki E, Lerner K, Roh D, Goldman SA (2001). Adenoviral brain-derived neurotrophic factor induces both neostriatal and olfactory neuronal recruitment from endogenous progenitor cells in the adult forebrain. *Journal of Neuroscience*.

[50] Jin K, Zhu Y, Sun Y, Mao XO, Xie L, Greenberg DA (2002). Vascular endothelial growth factor (VEGF) stimulates neurogenesis in vitro and in vivo. *Proceedings of the National Academy of Sciences of the United States of America*.

[51] Wang L, Zhang Z, Wang Y, Zhang R, Chopp M (2004). Treatment of stroke with erythropoietin enhances neurogenesis and angiogenesis and improves neurological function in rats. *Stroke*.

[52] Kimura A, Ohmori T, Kashiwakura Y (2008). Antagonism of sphingosine 1-phosphate receptor-2 enhances migration of neural progenitor cells toward an area of brain infarction. *Stroke*.

[53] Rueger MA, Muesken S, Walberer M (2012). Effects of minocycline on endogenous neural stem cells after experimental stroke. *Neuroscience*.

[54] Ehrenreich H, Weissenborn K, Prange H (2009). Recombinant human erythropoietin in the treatment of acute ischemic stroke. *Stroke*.

[55] Garzón-Muvdi T, Quiñones-Hinojosa A (2010). Neural stem cell niches and homing: recruitment and integration into functional tissues. *ILAR Journal*.

[56] Lindvall O, Kokaia Z (2011). Stem cell research in stroke: how far from the clinic?. *Stroke*.

[57] Hori J, Ng TF, Shatos M, Klassen H, Streilein JW, Young MJ (2003). Neural progenitor cells lack immunogenicity and resist destruction as allografts. *Stem Cells*.

[58] Yin L, Fu S-L, Shi G-Y (2008). Expression and regulation of major histocompatibility complex on neural stem cells and their lineages. *Stem Cells and Development*.

[59] Laguna Goya R, Busch R, Mathur R, Coles AJ, Barker RA (2011). Human fetal neural precursor cells can up-regulate MHC class I and class II expression and elicit CD4 and CD8 T cell proliferation. *Neurobiology of Disease*.

[60] Jablonska A, Janowski M, Lukomska B (2013). Different methods of immunosuppresion do not prolong the survival of human cord blood-derived neural stem cells transplanted into focal brain-injured immunocompetent rats. *Acta Neurobiologiae Experimentalis*.

[61] Takahashi K, Yasuhara T, Shingo T (2008). Embryonic neural stem cells transplanted in middle cerebral artery occlusion model of rats demonstrated potent therapeutic effects, compared to adult neural stem cells. *Brain Research*.

[62] Jensen MB, Yan H, Krishnaney-Davison R, Al Sawaf A, Zhang SC Survival and differentiation of transplanted neural stem cells derived from human induced pluripotent stem cells in a rat stroke model. *Journal of Stroke and Cerebrovascular Diseases*.

[63] Zhu W, Mao Y, Zhao Y (2005). Transplantation of vascular endothelial growth factor-transfected neural stem cells into the rat brain provides neuroprotection after transient focal cerebral ischemia. *Neurosurgery*.

[64] Zhang Z-H, Wang R-Z, Wang R-Z (2008). Transplantation of neural stem cells modified by human neurotrophin-3 promotes functional recovery after transient focal cerebral ischemia in rats. *Neuroscience Letters*.

[65] Jenny B, Kanemitsu M, Tsupykov O (2009). Fibroblast growth factor-2 overexpression in transplanted neural progenitors promotes perivascular cluster formation with a neurogenic potential. *Stem Cells*.

[66] Chen B, Gao X-Q, Yang C-X (2009). Neuroprotective effect of grafting GDNF gene-modified neural stem cells on cerebral ischemia in rats. *Brain Research*.

[67] Lee HJ, Lim IJ, Lee MC, Kim SU (2010). Human neural stem cells genetically modified to overexpress brain-derived neurotrophic factor promote functional recovery and neuroprotection in a mouse stroke model. *Journal of Neuroscience Research*.

[68] Wu W, Chen X, Hu C, Li J, Yu Z, Cai W (2010). Transplantation of neural stem cells expressing hypoxia-inducible factor-1*α* (HIF-1*α*) improves behavioral recovery in a rat stroke model. *Journal of Clinical Neuroscience*.

[69] Kelly S, Bliss TM, Shah AK (2004). Transplanted human fetal neural stem cells survive, migrate, and differentiate in ischemic rat cerebral cortex. *Proceedings of the National Academy of Sciences of the United States of America*.

[70] Chu K, Kim M, Park K-I (2004). Human neural stem cells improve sensorimotor deficits in the adult rat brain with experimental focal ischemia. *Brain Research*.

[71] Chu K, Park K-I, Lee S-T (2005). Combined treatment of vascular endothelial growth factor and human neural stem cells in experimental focal cerebral ischemia. *Neuroscience Research*.

[72] Zhang P, Li J, Liu Y (2009). Human neural stem cell transplantation attenuates apoptosis and improves neurological functions after cerebral ischemia in rats. *Acta Anaesthesiologica Scandinavica*.

[73] Stroemer P, Patel S, Hope A, Oliveira C, Pollock K, Sinden J (2009). The neural stem cell line CTX0E03 promotes behavioral recovery and endogenous neurogenesis after experimental stroke in a dose-dependent fashion. *Neurorehabilitation and Neural Repair*.

[74] Jin K, Mao X, Xie L (2010). Transplantation of human neural precursor cells in Matrigel scaffolding improves outcome from focal cerebral ischemia after delayed postischemic treatment in rats. *Journal of Cerebral Blood Flow and Metabolism*.

[75] Minnerup J, Kim JB, Schmidt A (2011). Effects of neural progenitor cells on sensorimotor recovery and endogenous repair mechanisms after photothrombotic stroke. *Stroke*.

[76] Sun C, Zhang H, Li J (2010). Modulation of the major histocompatibility complex by neural stem cell-derived neurotrophic factors used for regenerative therapy in a rat model of stroke. *Journal of Translational Medicine*.

[77] Bacigaluppi M, Pluchino S, Jametti LP (2009). Delayed post-ischaemic neuroprotection following systemic neural stem cell transplantation involves multiple mechanisms. *Brain*.

[78] Jeong S-W, Chu K, Jung K-H, Kim SU, Kim M, Roh J-K (2003). Human neural stem cell transplantation promotes functional recovery in rats with experimental intracerebral hemorrhage. *Stroke*.

[79] Yu H, Cao B, Feng M (2010). Combinated transplantation of neural stem cells and collagen type I promote functional recovery after cerebral ischemia in rats. *Anatomical Record*.

[80] Zhang P, Li J, Liu Y (2011). Human embryonic neural stem cell transplantation increases subventricular zone cell proliferation and promotes peri-infarct angiogenesis after focal cerebral ischemia. *Neuropathology*.

[81] Gaudier M, Schuwirth BS, Westcott SL, Wigley DB (2007). Structural basis of DNA replication origin recognition by an ORC protein. *Science*.

[82] Imitola J, Raddassi K, Park KI (2004). Directed migration of neural stem cells to sites of CNS injury by the stromal cell-derived factor 1*α*/CXC chemokine receptor 4 pathway. *Proceedings of the National Academy of Sciences of the United States of America*.

[83] Mueller F-J, Serobyan N, Schraufstatter IU (2006). Adhesive interactions between human neural stem cells and inflamed human vascular endothelium are mediated by integrins. *Stem Cells*.

[84] Ohab JJ, Fleming S, Blesch A, Carmichael ST (2006). A neurovascular niche for neurogenesis after stroke. *Journal of Neuroscience*.

[85] Pluchino S, Quattrini A, Brambilla E (2003). Injection of adult neurospheres induces recovery in a chronic model of multiple sclerosis. *Nature*.

[86] Pluchino S, Zanotti L, Rossi B (2005). Neurosphere-derived multipotent precursors promote neuroprotection by an immunomodulatory mechanism. *Nature*.

[87] Sawamoto K, Wichterle H, Gonzalez-Perez O (2006). New neurons follow the flow of cerebrospinal fluid in the adult brain. *Science*.

[88] Andres RH, Choi R, Pendharkar AV (2011). The CCR2/CCL2 interaction mediates the transendothelial recruitment of intravascularly delivered neural stem cells to the ischemic brain. *Stroke*.

[89] Doeppner TR, Ewert TA, Tonges L (2012). Transduction of neural precursor cells with TAT-heat shock protein 70 chaperone: therapeutic potential against ischemic stroke after intrastriatal and systemic transplantation. *Stem Cells*.

[90] Darsalia V, Allison SJ, Cusulin C (2011). Cell number and timing of transplantation determine survival of human neural stem cell grafts in stroke-damaged rat brain. *Journal of Cerebral Blood Flow and Metabolism*.

[91] Kim D-E, Schellingerhout D, Ishii K, Shah K, Weissleder R (2004). Imaging of stem cell recruitment to ischemic infarcts in a murine model. *Stroke*.

[92] Guzman R, Bliss T, De Los Angeles A, Moseley M, Palmer T, Steinberg G (2008). Neural progenitor cells transplanted into the uninjured brain undergo targeted migration after stroke onset. *Journal of Neuroscience Research*.

[93] Riess P, Zhang C, Saatman KE (2002). Transplanted neural stem cells survive, differentiate, and improve neurological motor function after experimental traumatic brain injury. *Neurosurgery*.

[94] Zhu JM, Zhao YY, Chen SD, Zhang WH, Lou L, Jin X (2011). Functional recovery after transplantation of neural stem cells modified by brain-derived neurotrophic factor in rats with cerebral ischaemia. *Journal of International Medical Research*.

[95] Englund U, Björklund A, Wictorin K, Lindvall O, Kokaia M (2002). Grafted neural stem cells develop into functional pyramidal neurons and integrate into host cortical circuitry. *Proceedings of the National Academy of Sciences of the United States of America*.

[96] Nan Z, Grande A, Sanberg CD, Sanberg PR, Low WC (2005). Infusion of human umbilical cord blood ameliorates neurologic deficits in rats with hemorrhagic brain injury. *Annals of the New York Academy of Sciences*.

[97] Schaller B, Andres RH, Huber AW (2005). Effect of GDNF on differentiation of cultured ventral mesencephalic dopaminergic and non-dopaminergic calretinin-expressing neurons. *Brain Research*.

[98] Harms KM, Li L, Cunningham LA (2010). Murine neural stem/progenitor cells protect neurons against ischemia by HIF-1alpha-regulated VEGF signaling. *PLoS ONE*.

[99] Andres RH, Horie N, Slikker W (2011). Human neural stem cells enhance structural plasticity and axonal transport in the ischaemic brain. *Brain*.

[100] Jin K, Xie L, Mao X (2011). Effect of human neural precursor cell transplantation on endogenous neurogenesis after focal cerebral ischemia in the rat. *Brain Research*.

[101] Jin K, Wang X, Xie L, Mao XO, Greenberg DA (2010). Transgenic ablation of doublecortin-expressing cells suppresses adult neurogenesis and worsens stroke outcome in mice. *Proceedings of the National Academy of Sciences of the United States of America*.

[102] Lee S-T, Chu K, Jung K-H (2008). Anti-inflammatory mechanism of intravascular neural stem cell transplantation in haemorrhagic stroke. *Brain*.

[103] Hassani Z, O'Reilly J, Pearse Y (2012). Human neural progenitor cell engraftment increases neurogenesis and microglial recruitment in the brain of rats with stroke. *PLoS ONE*.

[104] Jiang Q, Zheng GZ, Guang LD (2005). Investigation of neural progenitor cell induced angiogenesis after embolic stroke in rat using MRI. *NeuroImage*.

[105] Pluchino S, Martino G (2008). Neural stem cell-mediated immunomodulation: repairing the haemorrhagic brain. *Brain*.

[106] Friedenstein AJ, Piatetzky-Shapiro II, Petrakova KV (1966). Osteogenesis in transplants of bone marrow cells. *Journal of Embryology and Experimental Morphology*.

[107] Zou Z, Zhang Y, Hao L (2010). More insight into mesenchymal stem cells and their effects inside the body. *Expert Opinion on Biological Therapy*.

[108] Dominici M, Le Blanc K, Mueller I (2006). Minimal criteria for defining multipotent mesenchymal stromal cells. The International Society for Cellular Therapy position statement. *Cytotherapy*.

[109] Pittenger MF, Mackay AM, Beck SC (1999). Multilineage potential of adult human mesenchymal stem cells. *Science*.

[110] Kurozumi K, Nakamura K, Tamiya T (2004). BDNF gene-modified mesenchymal stem cells promote functional recovery and reduce infarct size in the rat middle cerebral artery occlusion model. *Molecular Therapy*.

[111] Liu H, Honmou O, Harada K (2006). Neuroprotection by PIGF gene-modified human mesenchymal stem cells after cerebral ischaemia. *Brain*.

[112] Toyama K, Honmou O, Harada K (2009). Therapeutic benefits of angiogenetic gene-modified human mesenchymal stem cells after cerebral ischemia. *Experimental Neurology*.

[113] Kurozumi K, Nakamura K, Tamiya T (2005). Mesenchymal stem cells that produce neurotrophic factors reduce ischemic damage in the rat middle cerebral artery occlusion model. *Molecular Therapy*.

[114] Ikeda N, Nonoguchi N, Ming ZZ (2005). Bone marrow stromal cells that enhanced fibroblast growth factor-2 secretion by herpes simplex virus vector improve neurological outcome after transient focal cerebral ischemia in rats. *Stroke*.

[115] Ding D-C, Shyu W-C, Chiang M-F (2007). Enhancement of neuroplasticity through upregulation of *β*1-integrin in human umbilical cord-derived stromal cell implanted stroke model. *Neurobiology of Disease*.

[116] Chen J, Li Y, Wang L, Lu M, Zhang X, Chopp M (2001). Therapeutic benefit of intracerebral transplantation of bone marrow stromal cells after cerebral ischemia in rats. *Journal of the Neurological Sciences*.

[117] Xin H, Li Y, Shen LH (2010). Increasing tPa activity in astrocytes induced by multipotent mesenchymal stromal cells facilitate neurite outgrowth after stroke in the mouse. *PLoS ONE*.

[118] Shen LH, Xin H, Li Y (2011). Endogenous tissue plasminogen activator mediates bone marrow stromal cell-induced neurite remodeling after stroke in mice. *Stroke*.

[119] Leu S, Lin Y-C, Yuen C-M (2010). Adipose-derived mesenchymal stem cells markedly attenuate brain infarct size and improve neurological function in rats. *Journal of Translational Medicine*.

[120] Li Y, Chen J, Chen XG (2002). Human marrow stromal cell therapy for stroke in rat: neurotrophins and functional recovery. *Neurology*.

[121] Honma T, Honmou O, Iihoshi S (2006). Intravenous infusion of immortalized human mesenchymal stem cells protects against injury in a cerebral ischemia model in adult rat. *Experimental Neurology*.

[122] Onda T, Honmou O, Harada K, Houkin K, Hamada H, Kocsis JD (2008). Therapeutic benefits by human mesenchymal stem cells (hMSCs) and Ang-1 gene-modified hMSCs after cerebral ischemia. *Journal of Cerebral Blood Flow and Metabolism*.

[123] Ukai R, Honmou O, Harada K, Houkin K, Hamada H, Kocsis JD (2007). Mesenchymal stem cells derived from peripheral blood protects against ischemia. *Journal of Neurotrauma*.

[124] Omori Y, Honmou O, Harada K, Suzuki J, Houkin K, Kocsis JD (2008). Optimization of a therapeutic protocol for intravenous injection of human mesenchymal stem cells after cerebral ischemia in adult rats. *Brain Research*.

[125] Bao X, Feng M, Wei J (2011). Transplantation of Flk-1+ human bone marrow-derived mesenchymal stem cells promotes angiogenesis and neurogenesis after cerebral ischemia in rats. *European Journal of Neuroscience*.

[126] Li Y, Chen J, Wang L, Lu M, Chopp M (2001). Treatment of stroke in rat with intracarotid administration of marrow stromal cells. *Neurology*.

[127] Koh S-H, Kim KS, Choi MR (2008). Implantation of human umbilical cord-derived mesenchymal stem cells as a neuroprotective therapy for ischemic stroke in rats. *Brain Research*.

[128] Li J, Zhu H, Liu Y (2010). Human mesenchymal stem cell transplantation protects against cerebral ischemic injury and upregulates interleukin-10 expression in Macaca fascicularis. *Brain Research*.

[129] Yang M, Wei X, Li J, Heine LA, Rosenwasser R, Iacovitti L (2010). Changes in host blood factors and brain glia accompanying the functional recovery after systemic administration of bone marrow stem cells in ischemic stroke rats. *Cell Transplantation*.

[130] Keimpema E, Fokkens MR, Nagy Z (2009). Early transient presence of implanted bone marrow stem cells reduces lesion size after cerebral ischaemia in adult rats. *Neuropathology and Applied Neurobiology*.

[131] Pavlichenko N, Sokolova I, Vijde S (2008). Mesenchymal stem cells transplantation could be beneficial for treatment of experimental ischemic stroke in rats. *Brain Research*.

[132] Ohtaki H, Ylostalo JH, Foraker JE (2008). Stem/progenitor cells from bone marrow decrease neuronal death in global ischemia by modulation of inflammatory/immune responses. *Proceedings of the National Academy of Sciences of the United States of America*.

[133] Zhao M-Z, Nonoguchi N, Ikeda N (2006). Novel therapeutic strategy for stroke in rats by bone marrow stromal cells and ex vivo HGF gene transfer with HSV-1 vector. *Journal of Cerebral Blood Flow and Metabolism*.

[134] Zhang J, Li Y, Chen J (2004). Expression of insulin-like growth factor 1 and receptor in ischemic rats treated with human marrow stromal cells. *Brain Research*.

[135] Zacharek A, Shehadah A, Chen J (2010). Comparison of bone marrow stromal cells derived from stroke and normal rats for stroke treatment. *Stroke*.

[136] Wei L, Fraser JL, Lu Z-Y, Hu X, Yu SP (2012). Transplantation of hypoxia preconditioned bone marrow mesenchymal stem cells enhances angiogenesis and neurogenesis after cerebral ischemia in rats. *Neurobiology of Disease*.

[137] Wakabayashi K, Nagai A, Sheikh AM (2010). Transplantation of human mesenchymal stem cells promotes functional improvement and increased expression of neurotrophic factors in a rat focal cerebral ischemia model. *Journal of Neuroscience Research*.

[138] Tsai L-K, Wang Z, Munasinghe J, Leng Y, Leeds P, Chuang D-M (2011). Mesenchymal stem cells primed with valproate and lithium robustly migrate to infarcted regions and facilitate recovery in a stroke model. *Stroke*.

[139] Kranz A, Wagner D-C, Kamprad M (2010). Transplantation of placenta-derived mesenchymal stromal cells upon experimental stroke in rats. *Brain Research*.

[140] Gutierrez-Fernandez M, Rodriguez-Frutos B, Ramos-Cejudo J (2013). Effects of intravenous administration of allogenic bone marrow- and adipose tissue-derived mesenchymal stem cells on functional recovery and brain repair markers in experimental ischemic stroke. *Stem Cell Research & Therapy*.

[141] Delcroix GJ-R, Schiller PC, Benoit J-P, Montero-Menei CN (2010). Adult cell therapy for brain neuronal damages and the role of tissue engineering. *Biomaterials*.

[142] Fiedler J, Leucht F, Waltenberger J, Dehio C, Brenner RE (2005). VEGF-A and PlGF-1 stimulate chemotactic migration of human mesenchymal progenitor cells. *Biochemical and Biophysical Research Communications*.

[143] Wang L, Li Y, Chen X (2002). MCP-1, MIP-1, IL-8 and ischemic cerebral tissue enhance human bone marrow stromal cell migration in interface culture. *Hematology*.

[144] Bhasin A, Padma Srivastava MV, Mohanty S, Bhatia R, Kumaran SS, Bose S (2013). Stem cell therapy: a clinical trial of stroke. *Clinical Neurology and Neurosurgery*.

[145] Shen LH, Li Y, Chen J (2007). One-year follow-up after bone marrow stromal cell treatment in middle-aged female rats with stroke. *Stroke*.

[146] Mezey E, Chandross KJ, Harta G, Maki RA, McKercher SR (2000). Turning blood into brain: cells bearing neuronal antigens generated in vivo from bone marrow. *Science*.

[147] Mezey É, Key S, Vogelsang G, Szalayova I, David Lange G, Crain B (2003). Transplanted bone marrow generates new neurons in human brains. *Proceedings of the National Academy of Sciences of the United States of America*.

[148] Zhao L-R, Duan W-M, Reyes M, Keene CD, Verfaillie CM, Low WC (2002). Human bone marrow stem cells exhibit neural phenotypes and ameliorate neurological deficits after grafting into the ischemic brain of rats. *Experimental Neurology*.

[149] Fox LE, Shen J, Ma K (2010). Membrane properties of neuron-like cells generated from adult human bone-marrow-derived mesenchymal stem cells. *Stem Cells and Development*.

[150] Pacary E, Legros H, Valable S (2006). Synergistic effects of CoCl2 and ROCK inhibition on mesenchymal stem cell differentiation into neuron-like cells. *Journal of Cell Science*.

[151] Wislet-Gendebien S, Hans G, Leprince P, Rigo J-M, Moonen G, Rogister B (2005). Plasticity of cultured mesenchymal stem cells: switch from nestin-positive to excitable neuron-like phenotype. *Stem Cells*.

[152] Paul G, Özen I, Christophersen NS (2012). The adult human brain harbors multipotent perivascular mesenchymal stem cells. *PLoS ONE*.

[153] Chen J, Li Y, Katakowski M (2003). Intravenous bone marrow stromal cell therapy reduces apoptosis and promotes endogenous cell proliferation after stroke in female rat. *Journal of Neuroscience Research*.

[154] Chen J, Li Y, Wang L, Lu M, Chopp M (2002). Caspase inhibition by Z-VAD increases the survival of grafted bone marrow cells and improves functional outcome after MCAo in rats. *Journal of the Neurological Sciences*.

[155] Chopp M, Li Y, Zhang J (2008). Plasticity and remodeling of brain. *Journal of the Neurological Sciences*.

[156] Chen J, Zhang ZG, Li Y (2003). Intravenous administration of human bone marrow stromal cells induces angiogenesis in the ischemic boundary zone after stroke in rats. *Circulation Research*.

[157] Chen J, Chopp M (2006). Neurorestorative treatment of stroke: cell and pharmacological approaches. *NeuroRx*.

[158] Chen X, Li Y, Wang L (2002). Ischemic rat brain extracts induce human marrow stromal cell growth factor production. *Neuropathology*.

[159] Ghasemi H, Ghazanfari T, Yaraee R (2009). Evaluation of relationship between the serum levels of inflammatory mediators and ocular injuries induced by sulfur mustard: Sardasht-Iran Cohort Study. *International Immunopharmacology*.

[160] Guo F, Lv S, Lou Y (2012). Bone marrow stromal cells enhance the angiogenesis in ischaemic cortex after stroke: involvement of notch signalling. *Cell Biology International*.

[161] Schäbitz W-R, Steigleder T, Cooper-Kuhn CM (2007). Intravenous brain-derived neurotrophic factor enhances poststroke sensorimotor recovery and stimulates neurogenesis. *Stroke*.

[162] Scheibe F, Ladhoff J, Huck J (2012). Immune effects of mesenchymal stromal cells in experimental stroke. *Journal of Cerebral Blood Flow and Metabolism*.

[163] Yoo SW, Chang DY, Lee HS (2013). Immune following suppression mesenchymal stem cell transplantation in the ischemic brain is mediated by TGF-beta. *Neurobiology of Disease*.

[164] Honmou O, Houkin K, Matsunaga T (2011). Intravenous administration of auto serum-expanded autologous mesenchymal stem cells in stroke. *Brain*.

[165] Jiang Y, Zhu W, Zhu J, Wu L, Xu G, Liu X Feasibility of delivering mesenchymal stem cells via catheter to the proximal end of lesion artery in patients with stroke in the territory of middle cerebral artery. *Cell Transplantation*.

[166] Lappalainen RS, Narkilahti S, Huhtala T (2008). The SPECT imaging shows the accumulation of neural progenitor cells into internal organs after systemic administration in middle cerebral artery occlusion rats. *Neuroscience Letters*.

[167] Levitt JM, Lodhi IJ, Nguyen PK (2003). Low-dose sulfur mustard primes oxidative function and induces apoptosis in human polymorphonuclear leukocytes. *International Immunopharmacology*.

[168] Mitkari B, Kerkela E, Nystedt J (2013). Intra-arterial infusion of human bone marrow-derived mesenchymal stem cells results in transient localization in the brain after cerebral ischemia in rats. *Experimental Neurology*.

[169] White BC, Sullivan JM, DeGracia DJ (2000). Brain ischemia and reperfusion: molecular mechanisms of neuronal injury. *Journal of the Neurological Sciences*.

[170] Lee HJ, Kim KS, Park IH, Kim SU (2007). Human neural stem cells over-expressing VEGF provide neuroprotection, angiogenesis and functional recovery in mouse stroke model. *PLoS ONE*.

[171] Lee HJ, Kim MK, Kim HJ, Kim SU (2009). Human neural stem cells genetically modified to overexpress Akt1 provide neuroprotection and functional improvement in mouse stroke model. *PLoS ONE*.

[172] Sakata H, Narasimhan P, Niizuma K, Maier CM, Wakai T, Chan PH (2012). Interleukin 6-preconditioned neural stem cells reduce ischaemic injury in stroke mice. *Brain*.

[173] Modo M, Mellodew K, Cash D (2004). Mapping transplanted stem cell migration after a stroke: a serial, in vivo magnetic resonance imaging study. *NeuroImage*.

[174] Shyu W-C, Chen C-P, Lin S-Z, Lee Y-J, Li H (2007). Efficient tracking of non-iron-labeled mesenchymal stem cells with serial MRI in chronic stroke rats. *Stroke*.

[175] Bulte JWM, Douglas T, Witwer B (2001). Magnetodendrimers allow endosomal magnetic labeling and in vivo tracking of stem cells. *Nature Biotechnology*.

[176] Rudelius M, Daldrup-Link HE, Heinzmann U (2003). Highly efficient paramagnetic labelling of embryonic and neuronal stem cells. *European Journal of Nuclear Medicine and Molecular Imaging*.

[177] Ward RJ, Wilmet S, Legssyer R, Crichton RR (2002). The influence of iron homoeostasis on macrophage function. *Biochemical Society Transactions*.

[178] Khalil MM, Tremoleda JL, Bayomy TB, Gsell W (2011). Molecular SPECT imaging: an overview. *International Journal of Molecular Imaging*.

[179] Lalu MM, McIntyre L, Pugliese C (2012). Safety of cell therapy with mesenchymal stromal cells (SafeCell): a systematic review and meta-analysis of clinical trials. *PLoS ONE*.

[180] Nomura T, Honmou O, Harada K, Houkin K, Hamada H, Kocsis JD (2005). I.v. infusion of brain-derived neurotrophic factor gene-modified human mesenchymal stem cells protects against injury in a cerebral ischemia model in adult rat. *Neuroscience*.

[181] Horita Y, Honmou O, Harada K, Houkin K, Hamada H, Kocsis JD (2006). Intravenous administration of glial cell line-derived neurotrophic factor gene-modified human mesenchymal stem cells protects against injury in a cerebral ischemia model in the adult rat. *Journal of Neuroscience Research*.

[182] Cho G-W, Koh S-H, Kim M-H (2010). The neuroprotective effect of erythropoietin-transduced human mesenchymal stromal cells in an animal model of ischemic stroke. *Brain Research*.

[183] Ding J, Cheng Y, Gao S, Chen J (2011). Effects of nerve growth factor and Noggin-modified bone marrow stromal cells on stroke in rats. *Journal of Neuroscience Research*.

